# Structural and practical identifiability analysis in bioengineering: a beginner’s guide

**DOI:** 10.1186/s13036-024-00410-x

**Published:** 2024-03-04

**Authors:** Linda Wanika, Joseph R. Egan, Nivedhitha Swaminathan, Carlos A. Duran-Villalobos, Juergen Branke, Stephen Goldrick, Mike Chappell

**Affiliations:** 1 School of Engineering, University of Warwick, Coventry, CV4 7AL United Kingdom; 2 Department of Biochemical Engineering, University College London, London, United Kingdom; 3 Department of Electrical and Electronic Engineering, University of Manchester, Manchester, United Kingdom; 4 Warwick Business School, University of Warwick, Coventry, United Kingdom

## Abstract

Advancements in digital technology have brought modelling to the forefront in many disciplines from healthcare to architecture. Mathematical models, often represented using parametrised sets of ordinary differential equations, can be used to characterise different processes. To infer possible estimates for the unknown parameters, these models are usually calibrated using associated experimental data. Structural and practical identifiability analyses are a key component that should be assessed prior to parameter estimation. This is because identifiability analyses can provide insights as to whether or not a parameter can take on single, multiple, or even infinitely or countably many values which will ultimately have an impact on the reliability of the parameter estimates. Also, identifiability analyses can help to determine whether the data collected are sufficient or of good enough quality to truly estimate the parameters or if more data or even reparameterization of the model is necessary to proceed with the parameter estimation process. Thus, such analyses also provide an important role in terms of model design (structural identifiability analysis) and the collection of experimental data (practical identifiability analysis). Despite the popularity of using data to estimate the values of unknown parameters, structural and practical identifiability analyses of these models are often overlooked. Possible reasons for non-consideration of application of such analyses may be lack of awareness, accessibility, and usability issues, especially for more complicated models and methods of analysis. The aim of this study is to introduce and perform both structural and practical identifiability analyses in an accessible and informative manner via application to well established and commonly accepted bioengineering models. This will help to improve awareness of the importance of this stage of the modelling process and provide bioengineering researchers with an understanding of how to utilise the insights gained from such analyses in future model development.

## Introduction

The use of mathematical models has risen considerably over the last decade, with many industries utilising models to represent processes that are of practical importance in today’s modern world [1–3]. In bioengineering, models have been developed e.g. to characterise protein regulation, metabolic changes in cells and pharmacokinetic and pharmacodynamic processes [4–7]. Representing these processes in a mathematical form has further advanced our knowledge and understanding of fundamental mechanisms and dynamics involved in these processes across many sectors, eventually reducing reliance on animal testing, improving the optimisation of process manufacture, and reducing in cost and time expenses [8–11].

Typically, first principle models in bioengineering often comprise sets of parameterised (nonlinear) ordinary differential equations (ODEs). These equations are often derived through application of the fundamental law of mass balance to represent changes that occur over time in the highlighted states [12]. These states and ODEs generally have sets of parameters which need to be accounted for either through experimental insights or more commonly through “fitting” the model developed to available data.

The results generated by these methods may yield parameter estimates that provide model simulation outputs that are closely aligned with the data, however, they do not ascertain whether these estimates are the only estimates capable of capturing the data. An issue is that some unknown parameters within the given model may take on any value from an infinite number of possible values yet still permit good (visual) agreement between simulated model responses and the data and this decreases the reliability of such parameter estimates and the model itself.

Structural identifiability analysis (SIA) can be used to investigate whether parameters can take on unique, finite, or infinite (thus termed structurally unidentifiable) values for the given model outputs/observations, but such analysis is not commonly employed in many preliminary modelling processes [13–15]. SIA is used to determine whether a parameter can be uniquely or otherwise identified based on the postulated system of model ODEs, known input(s) and, in particular, model output(s) (as the number of measured outputs for the same ODE system can alter the identifiability of the model). Such analysis thus offers important support for experiment design as well as robust parameter estimation, especially in the case where unidentifiability has been established. Some parameters may also have strong correlations with others in the model, and such dependence may be determined from a SIA where only a certain parameter combination set can be identified rather than the individual parameters themselves. In this case a plausible model redesign would be to treat the parameter combination as one parameter via an appropriate model reparameterisation.

Despite the fundamental insights that can be gained from SIA and the importance of having a structurally identifiable model, especially where certain model parameters may have important physical or practical significance, several models have been published in the literature which have been shown to be structurally unidentifiable [14–16]. As structural identifiability is a prerequisite to experiment design and system identification/parameter estimation, It is crucially important to perform SIA prior to data collection, as the results obtained from SIA can be used to highlight which parameters need to be measured as well as how many outputs need to be observed. Moreover, the model can be redesigned if the SIA results deem the model to be structurally unidentifiable for the given experiments and proposed model structure.

Analysis as to whether unknown model parameters can be identified based not only on the postulated system model but also on the data that can be used to estimate these parameter values, a process known as practical identifiability analysis (PIA), is seemingly performed even less often [17]. Both forms of analysis should be employed as core components in the model building process. There are multiple methods and tools that are available for both SIA and PIA [14, 17, 18]. However, these tools can be challenging to access, utilise and in some instances be restrictive, which may be a reason for their lack of generic usage. For SIA, the Laplace transform approach, for example, is limited to linear models only [13, 14]. PIA approaches are present in the literature, however the tools that are available are limited and these studies typically express the fundamentals and practice for a specialised audience. Nevertheless, we advocate that both SIA and PIA should be attempted, or at the very least considered, every time that a model is being fitted to data with the aim of parameter estimation.

This study aims to provide an accessible step-by step process on both performing structural and practical identifiability analyses with application to models in bioengineering. Specifically, in the Methods section, both accessible and technical explanations of the approaches to performing structural (section "Structural identifiability analysis") and practical identifiability analysis (section "Practical identifiability analysis") will be given. Moreover, the tools that are used are applicable to both linear and non-linear models, steps on how to access and utilise these tools will be shown (section "Implementation of identifiability tools and associated codes"). The final Methods section (section "Models and data used for the analysis") will introduce three exemplar bioengineering models which will be subsequently analysed in the Results section.

## Methods

**Fig. 1:**
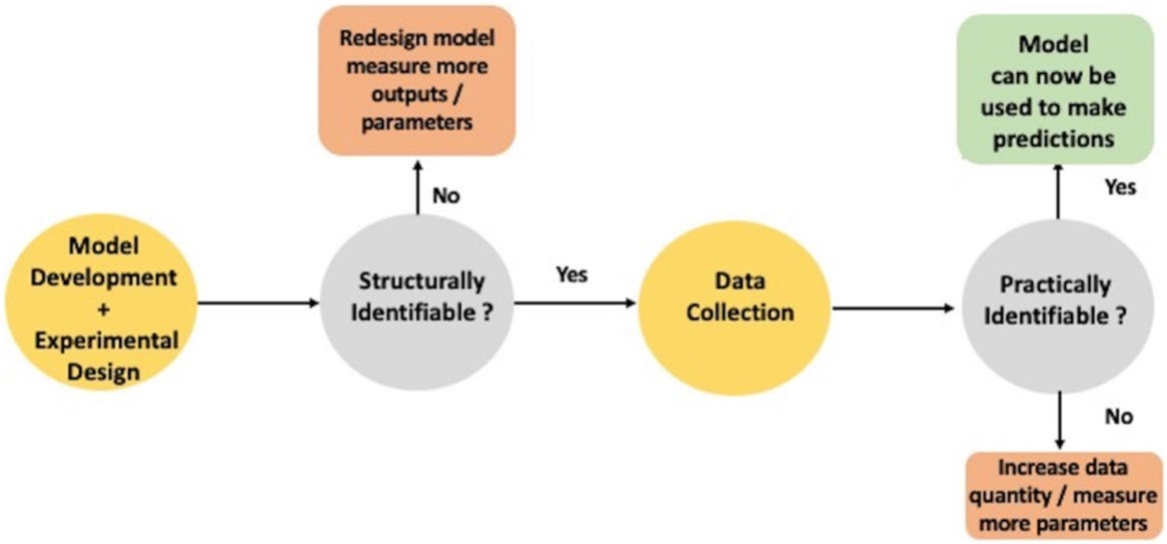
Brief overview of the model development process for predictive models

Figure 1 summarises the important steps that should be performed in order to develop a model that can be used reliably for making parameter predictions:

Both structural and practical identifiability analyses are required in order to develop a predictive model with increased reliability.

### Structural identifiability analysis

#### Accessible definition

For an intuitive approach to structural identifiability, consider the equations below:

1 $$y\left(t\right)=a\times 2$$

2 $$y\left(t\right)={a}^{2}\times 2$$

3 $$y\left(t\right)=a+b$$

where $a$ and $b$ are unknown parameters and $y\left(t\right)$, the measured output, is always known. Parameter $a$ in Eqn(1) can be identified as one unique solution because $y\left(t\right)$ can be divided by 2 to identify $a$. Parameter $a$ can also be identified in Eqn(2); however, this will result in two solutions; a negative value and a positive value, thus $a$ is said to be locally identifiable. Lastly, since neither of the parameters $a$ nor $b$ are known, Eqn(3) is said to be structurally unidentifiable. This is because an infinite number of possible values for $a$ and $b$ can give rise to the same response.

#### Technical definition

The basic form for the mathematical models to be considered is as follows:

4 $$M:\left\{\begin{array}{c} \dot{x}\left(t,p\right)=f\left(x\left(t\right),u\left(t\right),p\right),\\ y\left(t,p\right)=g\left(x\left(t\right),p\right),\\ {x}_{0}=x({t}_{0},p)\end{array}\right.$$

where $f$ and $g$ are vector functions of their arguments, $p\in {\mathbb{R}}^{p}$ is a *p*-dimensional vector of parameters, $x(t)\in {\mathbb{R}}^{n}$ is the *n*-dimensional state variable vector, $u(t)\in {\mathbb{R}}^{r}$ is the *r*-dimensional input vector and $y(t)\in {\mathbb{R}}^{m}$ are the *m*-dimensional measured outputs [15].

For model (4), a parameter ${p}_{i}$ is said to be identifiable if the following equation holds true:

5 $$y\left(t,p\right)=y\left(t,{p}^{*}\right)\Rightarrow{p}_{i}={p}_{i}^{*}$$

where ${p}^{*}$ is an alternative parameter vector. If Eqn(5) holds for any ${p}_{i}^{*}$, then ${p}_{i}$ is said to be globally identifiable. If Eqn(5) holds for a neighbourhood vector of ${p}_{i}^{*}$, then ${p}_{i}$ is said to be locally identifiable. Finally, if Eqn(5) does not hold true for any ${p}_{i}$ locally or globally then ${p}_{i}$ is said to be structurally unidentifiable. For a model to be deemed to be structurally identifiable, all its parameters must be at least locally identifiable.

#### Methods for performing structural identifiability analysis

There are multiple methods that can be used to perform SIA [14]. The two methods that will be used in this paper are the Taylor series approach and the Exact Arithmetic Rank (EAR) approach. The Taylor series approach is a simple concept to understand and a simple way to analyse the structural identifiability of parameters. The EAR approach has been developed into a freely available *MATHEMATICA* tool [19], which enables users to enter their system of ODEs, input(s), and output(s), and will determine if the system is at least locally identifiable. This tool also identifies which parameters need to be known (a *priori*) in order for the system to be identifiable [14, 20–23].

In the Taylor series approach, a 1-dimensional observation function $y\left(t\right)$ can be expanded as a Taylor series around a known time point, which in practice is usually a known initial condition when $t=0$:

6 $$y\left(t\right)=y\left(0\right)+{y}^{\prime}\left(0\right)\left(t\right)+\frac{{y}^{{\prime}{\prime}}(0)}{2!}{(t)}^{2}+\dots +\frac{{y}^{n}\left(0\right)}{n!}{\left(t\right)}^{n}+$$

where ${y}^{\prime}$ refers to the first derivative of $y$ and $n$ refers to the nth term in the Taylor series expansion. It is assumed that all derivatives that appear as coefficients in the Taylor series of the observed outputs are unique and measurable and are thus known [13, 14]. Therefore, it is possible to investigate the solutions for the unknown parameters within these coefficients. A single or multiple solution(s) (not infinite solutions) would indicate that the parameter is identifiable like Eqns (3-4). As the number of coefficients derived from the Taylor series expansion is infinite, for linear systems, there are limits which have been determined on the maximum number of coefficients that are needed in order to determine whether a model is at least locally identifiable [14]. For linear systems the maximum number of coefficients is $2n-1$ , where $n$ is the number of states. The same upper bound rule can be applied for non-linear systems, however, this is not a strict upper bound limit [14]. For example, if a linear system of ODEs comprises of two states then a third linearly independent coefficient would be the last coefficient that could be used in order to determine the structural identifiability of the system.

Like the Taylor series approach, the observation $y\left(t\right)$ in the EAR approach is also differentiated with respect to time. However, the derivatives here are based on partial derivatives with respect to the states, inputs, and the parameters. Once computed the Jacobian matrix is formed of these partial derivatives and its rank is then assessed [24]. A Jacobian with full rank would indicate that all the columns (i.e., the parameters) are linearly independent from each other which would suggest that the model is at least locally identifiable. A deficient matrix rank would suggest that some of the parameters are dependent on each other (which would make this model structurally unidentifiable) and would also provide information on the number of degrees of freedom required for the parameter set/model to be deemed at least locally identifiable.

To interpret this analysis in an intuitive way, consider Eqn(3). Parameters $a$ and $b$ are entirely dependent on each other, in other words any value that is placed for $a$, will also always change the value of $b$.

### Practical identifiability analysis

Structural identifiability only considers the structure of the system of ODEs, the model inputs and, in particular, what the measured outputs are. It does not consider how the outputs are measured, in other words the associated data. PIA aims to assess whether the parameters can be at least locally identified based on the system of ODEs and the associated experimental data. It is worth emphasising that a model needs to be structurally identifiable for it to be practically identifiable. While there are multiple methods for performing a PIA, this study will focus on the profile likelihood approach.

#### Profile likelihood analysis: technical explanation

In the profile likelihood approach, a particular parameter *p*_*i*_ is discretised using a step size (determined by many factors including the upper and lower boundary values of *p*_*i*_ and the initial *p*_*i*_ estimate) and the other parameters in the model (called nuisance parameters) are then reoptimised using the new values of *p*_*i*_ to fit to the data. The loglikelihood ratio is used to determine whether the new fit to the data is significantly different or improved with respect to the previous fit to the data (e.g. a model with *p*_*i*_(1) compared to a model with *p*_*i*_(2)) [25]. A likelihood based 95% CI threshold is generated based on the ${\chi }^{2}$ distribution and the mode’s number of degrees of freedom. If the 95% CI is finite, then the parameter is practically identifiable. If the 95% CI is infinite, then the parameter is practically unidentifiable.

#### Profile likelihood analysis: accessible explanation

**Fig. 2:**
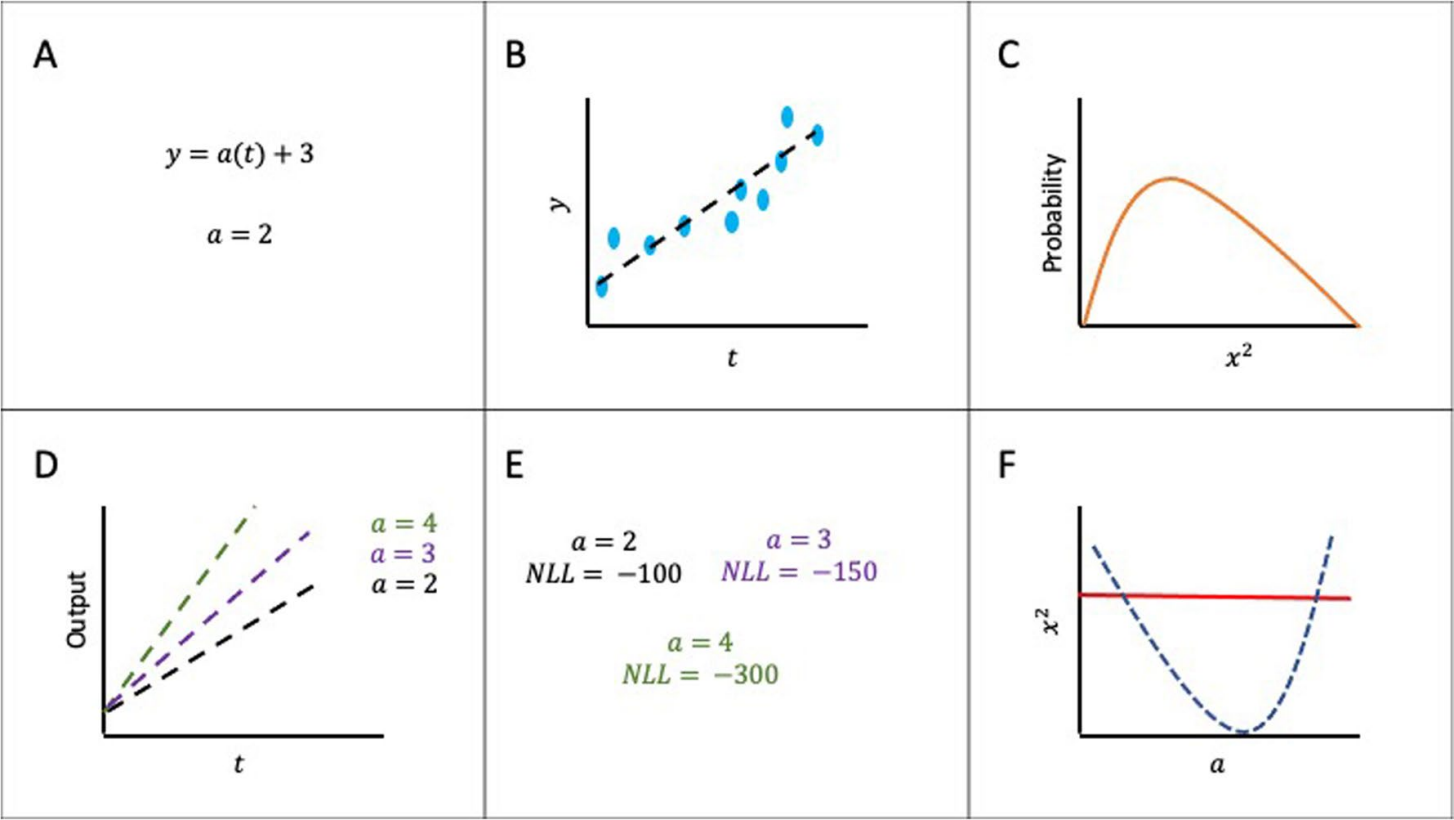
Simplistic overview of practical identifiability analysis using the profile likelihood process. NLL: negative log likelihood. **A** Example model with parameter estimate. **B** Example model fitting to observed data. **C** Chi- squared (${\chi }^{2}$) distribution (orange) for the associated model. **D** Model simulation based on different values for $a$. **E** Different negative loglikelihood values based on the different parameter estimates. F: profile likelihood of $a$. Note that some tools will use ${\chi }^{2}$ and negative loglikelihood interchangeably for the profile likelihood plots. Each of the steps are explained below

Figure 2 visualises the general steps for computing the profile likelihood for the parameters:

Figure 2A depicts a simple structurally identifiable model ($y=a\left(t\right)+3$) with one parameter ($a$) as well as an estimated value obtained for $a$. Figure 2B provides an exemplar fitting of the model (black dashed line) to the observed data (blue dots). Based on the observations and the number of parameters, the number of degrees of freedom for this particular model can be computed. In this example, the number of degrees of freedom is nine as there are 10 observations and one parameter that can vary. Figure 2C visualises a ${\chi }^{2}$ distribution which, together with the number of degrees of freedom for $y=a\left(t\right)+3$, can be used to obtain the 95% CI.

Figure 2D visualises a structurally identifiable parameter as the value of $a$ changes (depicted by the different coloured lines in the plot), the output response also changes. In parameter estimation, the data are assessed to evaluate how probable it is that the data can be generated based on the estimated parameter value, this statistic is known as the likelihood (usually, the goal is to minimise the negative log likelihood [26]). In Figure 2E, the different values used for $a$ generate different loglikelihood values. These values are compared to each other using the loglikelihood ratio to determine if there is a significant difference between them. Figure 2F provides a practically identifiable profile likelihood curve. The blue dashed line depicts the profile likelihood where different values for $a$ are compared to the previous estimates and generate different ${\chi }^{2}$ values (which are computed using the likelihood ratio - note that in some tools the y-axis is also referred to as the negative log likelihood as well as ${\chi }^{2}$). The red line depicts the 95% CI which the estimates need to cross on both sides in order for the parameter to be deemed as practically identifiable. If the 95% CIs are finite then the parameter is practically identifiable (e.g., $a$). However, if the 95% CIs are infinite then the parameter is practically unidentifiable.

### Implementation of identifiability tools and associated codes

Both the Taylor series approach and the EAR approach were performed in *MATHEMATICA*. The PIA was conducted in *MATLAB* using the data2dynamics toolkit [27, 28]. All of the relevant scripts and codes used for both structural and practical identifiability can be found in the associated Github repository [29].

### Models and data used for the analysis

All the models that have been used for this study have been published [30–32]. Data were extracted from the published reports using web plot digitizer, a tool that can be used to extract approximate data from figures [33]. Thus it is important to note that the PIA performed here is based on approximate data observations and not the actual data observations. The models have been rewritten in terms of notation for ease of interpreting the model states and parameters. All other assumptions are included in each of the relevant model sections.

#### Model 1

A two-state mRNA model comprising of reactions including transcription, mRNA degradation, translation and protein degradation was developed to assess the mechanism of mRNA and protein regulation during cooler temperatures [30]. The system of ODEs defining the model are as follows:

7 $$\frac{d(mRNA)}{dt}={k}_{1}-{k}_{2}\times mRNA$$

8 $$\frac{d(Protein)}{dt}={k}_{3}\times mRNA-{k}_{4}\times Protein$$

where $mRNA$ denotes mRNA activity and $Protein$ depicts protein activity. ${k}_{1}$ to ${k}_{4}$ refer to the transcription rate, mRNA degradation rate, translation rate and protein degradation rate, respectively. For this model, we assume that both model states are observed and thus measured and all parameters ${k}_{1}$ to ${k}_{4}$ are unknown. The initial conditions are assumed to be $mRNA \left(0\right)=2.5$ and $Protein \left(0\right)=6.5$, both with arbitrary units (a.u.).

#### Model 2

A three-state bioreactor model was developed to characterise cell growth in a bioreactor set-up [32]. The system of ODEs defining the model are given as follows:

9 $$\frac{d\left(Glucose\right)}{dt}={D}_{r}\times \left(GF - Glucose\right)-{r}_{glu}\times X$$

10 $$\frac{d(Lactate)}{dt}=-{D}_{r}\times {\text{Lactate}}+{r}_{lac}\times X$$

11 $$\frac{d\left(X\right)}{dt}= (({\mu }_{max}\times \frac{{\text{Glucose}}}{{km}_{glu}+{\text{Glucose}}}\times \frac{{ki}_{lac}}{{ki}_{lac}+{\left({\text{Lactate}}\right)}^{2}})- {D}_{r})\times X$$

where $Glucose$ and $Lactate$ refer to the concentrations of glucose and lactate in the bioreactor respectively. $X$ refers to the concentration of the cells in the bioreactor. ${D}_{r}$ is the dilution rate and *GF* is the glucose concentration in the feed, both of which are provided by the modeller. In the published study, ${D}_{r}$ is 0.033 hr^-1^ and $GF$ is 7mM. ${r}_{glu}$ and ${r}_{lac}$ refers to the consumption rate of glucose and production rate of lactate respectively. ${\mu }_{max}$ is the maximum growth rate of the cells. ${km}_{glu}$ and ${ki}_{lac}$ are the saturation constant of glucose and inhibition constant of lactate respectively. The original paper utilises this model along with an intracellular metabolic model in order to perform the predictions. However, given that only the ODEs are given, the analysis will be based on the ODE system alone. The following assumptions are made about the model: All states are observed and all parameters: {${r}_{glu}$, ${r}_{lac}$, ${\mu }_{max}$, ${km}_{glu}$, ${ki}_{lac}$} are unknown. The initial conditions for each of the state are as follows: $Glucose\left(0\right)=$ 1.01mM, $Lactate\left(0\right)=$ 3.98mM and $X\left(0\right)=$ 0.46 x10^6^ cells/mL.

#### Model 3

A two-state model was developed for erythroblast growth inhibition [31]. The system of ODEs defining the model is as follows:

12 $$\frac{d\left(X\right)}{dt}=\frac{{r}_{g}\times X}{\left(1+{e}^{{\text{a}}\times \left({\text{I}}-{\text{b}}\right)}\right)}$$

13 $$\frac{d\left(I\right)}{dt}=\frac{{r}_{g}\times X}{\left(1+{e}^{{\text{a}}\times \left({\text{I}}-{\text{b}}\right)}\right)}-{r}_{d}\times {\text{I}}$$

Where $X$ and $I$ refer to concentration of the cells and the inhibitor concentration respectively. ${r}_{g}$ and ${r}_{d}$ refer to the growth rate and the inhibitor decay rate respectively. ${\text{a}}$ and $b$ refer to the inhibitory sensitivity and threshold respectively. This model has been adapted from model 3 of the original study and assumes that only $X$ is observed. While in the original model the exponent term was ${e}^{{\text{a}}\times \left({\text{b}}-{\text{I}}\right)}$, the parameter estimates provided in the article match with the model system presented above. The parameters ${k}_{s}$ and ${k}_{c}$ were replaced by ${\text{a}}$ and $b$ respectively to dissociate them from being rate parameters. All parameters {${r}_{g}$, ${r}_{d}$, $a$, $b$} are assumed to be unknown. The initial conditions are assumed to be $X \left(0\right)=$ 4.45x10^6^ cells/mL*,* and $I \left(0\right)=$ 0x10^6^ cells/mL.

To utilise the EAR tool, this system of ODEs will need to be simplified (in order for it to align with software requirements that the model is polynomial in form) by introducing a new state to replace the exponential term. This can be performed as follows:

14 $${e}^{{\text{a}}\times \left({\text{I}}-{\text{b}}\right)}={e}^{-{\text{a}}\times {\text{b}}}\times {e}^{{\text{a}}\times {\text{I}}}$$

${e}^{-{\text{a}}\times {\text{b}}}$ can be replaced by a parameter ${r}_{ab}$ and ${e}^{{\text{a}}\times {\text{I}}}$ can be replaced by a new state termed $\varphi$. A differential equation defining this new state also needs to be derived to yield the augmented model to have the required state space form, thus the new augmented system of ODEs for model 3 is given by:

15 $$\frac{d(X)}{dt}=\frac{{r}_{g}\times X}{\left(1+{r}_{ab}\times \varphi \right)}$$

16 $$\frac{d\left(I\right)}{dt}=\frac{{r}_{g}\times X}{\left(1+{r}_{ab}\times \varphi \right)}-{r}_{d}\times {\text{I}}$$

17 $$\frac{d\left(\varphi \right)}{dt}=\varphi \times {\text{a}}\times (\frac{{r}_{g}\times X}{\left(1+{r}_{ab}\times \varphi \right)}-{r}_{d}\times {\text{I}})$$

The initial condition for $\varphi$ is 1 a.u. and this state is also not observed.

## Results

The SIA of model 1 is described in section "Model 1", The SIA, the model parameter estimates and the PIA for model 2 is described in section "Model 2" and for model 3, in section "Model 3". There are no associated experimental data with the mRNA model thus PIA is only performed on models 2 and 3. For the profile likelihood plots the thresholds are based on the thresholds values that are displayed for each of the profile likelihood plots (which are generated using the Data2Dynamics tool). While the Data2Dynamics results do provide a value for the threshold (known as “merit”), the value that is given is not always the same as the threshold that the tool automatically displays in the plots. For users who simply wish to utilise the plots generated by the Data2Dynamics tool, this is not an issue. However for users who wish to extract the data and plot in other software, then the threshold that is marked on the plots should be used.

### Model 1

#### Structural identifiability results

To understand how the states can be evaluated at $t=0$, the first three derivatives for $mRNA(t)$ and $Protein(t)$ are described as follows:

18 $$mRN{A}^{\prime}\left(t\right)= {k}_{1}-{k}_{2}\times mRNA(t)$$

19 $$mRN{A}^{\mathrm{^{\prime}}\mathrm{^{\prime}}}\left(t\right)= -{k}_{2}\times ({k}_{1}-{k}_{2}\times mRNA(t))$$

20 $$mRN{A}^{{\prime}{\prime}{\prime}}\left(t\right)= {{k}_{2}}^{2}\times ({k}_{1}-{k}_{2}\times mRNA(t))$$

21 $$Protein^{\prime}(t)={k}_{3}\times mRNA(t)-{k}_{4}\times Protein(t)$$

22 $$Protei{n}^{{\prime}{\prime}}\left(t\right)={k}_{3}\times \left({k}_{1}-{k}_{2}\times mRNA\left(t\right)\right)-{k}_{4}\times ({k}_{3}\times mRNA\left(t\right)-{k}_{4}\times Protein(t))$$

23 $$Protei{n}^{{\prime}{\prime}{\prime}}\left(t\right)=-{k}_{2}\times {k}_{3}\times \left({k}_{1}-{k}_{2}\times mRNA\left(t\right)\right)-{k}_{4}\times ({k}_{3}\times \left({k}_{1}-{k}_{2}\times mRNA\left(t\right)\right)-{k}_{4}\times \left({k}_{3}\times mRNA\left(t\right)-{k}_{4}\times Protein\left(t\right))\right)$$

Replacing $t$ with 0 in $mRNA(t)$ and $Protein(t)$ changes these terms to the initial conditions of $mRNA$ and $Protein$ respectively and can be used to provide the Taylor series coefficients for the analysis.

The ascending coefficients for the derivatives of the observations need to be generated, thus $mRNA$ and $Protein$ can be differentiated as follows when evaluated at $t=0$:

24 $$mRNA\left(0\right)=2.5$$

25 $$mRN{A}^{\prime}\left(0\right)= {k}_{1}-{k}_{2}\times 2.5$$

26 $$mRN{A}^{{\prime}{\prime}}\left(0\right)= -{k}_{2}\times ({k}_{1}-{k}_{2}\times 2.5 )$$

27 $$mRN{A}^{\mathrm{^{\prime}}\mathrm{^{\prime}}\mathrm{^{\prime}}}\left(0\right)= \left({k}_{1}-{k}_{2}\times 2.5 \right)\times {{k}_{2}}^{2}$$

28 $$Protein\left(0\right)=6.5$$

29 $$Protein^{\prime}\left(0\right)= {k}_{3}\times 2.5-{k}_{4}\times 6.5$$

30 $$Protei{n}^{{\prime}{\prime}}(0)= {k}_{3}\times \left({k}_{1}-{k}_{2}\times 2.5 \right)-{k}_{4}\times ({k}_{3}\times 2.5-{k}_{4}\times 6.5 )$$

31 $$Protei{n}^{{\prime}{\prime}{\prime}}\left(0\right)=-\left({k}_{1}-2.5\times {k}_{2}\right)\times {k}_{2}\times {k}_{3}-{k}_{4}\times (({k}_{1}-2.5\times {k}_{2})\times {k}_{3}-\left(2.5\times {k}_{3}-6.5\times {k}_{4}\right)\times {k}_{4}$$

Recall that all these coefficients are assumed to be unique for these observations and known. The following steps can then be used to identify all of the model parameters:

1) The parameter ${k}_{2}$ can be identified from Eqns (25-26) by substituting Eqn(25) into Eqn(26) which leaves ${k}_{2}$ as the only unknown parameter.

32 $$newmRN{A}^{{\prime}{\prime}}\left(0\right)= -{k}_{2}\times mRNA^{\prime}(0)$$

2) Since ${k}_{2}$ is now identifiable, ${k}_{1}$ can be identified from Eqn(25).

3)  ${k}_{3}$ can be solved and replaced using Eqn(29).

33 $${k}_{3}=2.6\times {k}_{4}+0.4\times Protein^{\prime}\left(0\right)$$

4) The new solution for ${k}_{3}$ can be used in Eqn(30) which leaves ${k}_{4}$ as the only unknown parameter and thus ${k}_{4}$ is identifiable.

5) With ${k}_{4}$, being identified ${k}_{3}$ can now be identified from Eqn(33).

Since all parameters have now been identified and shown to have unique solutions in terms of the known Taylor series coefficients, the mRNA model is therefore structurally uniquely identifiable based on the assumption that both $mRNA$ and $Protein$ are observed. If only $mRNA$ was observed, then model 1 would be structurally unidentifiable. Knowledge of ${k}_{3}$ and ${k}_{4}$ would be needed to make model 1 identifiable if only $mRNA$ was observed.

**Fig. 3:**
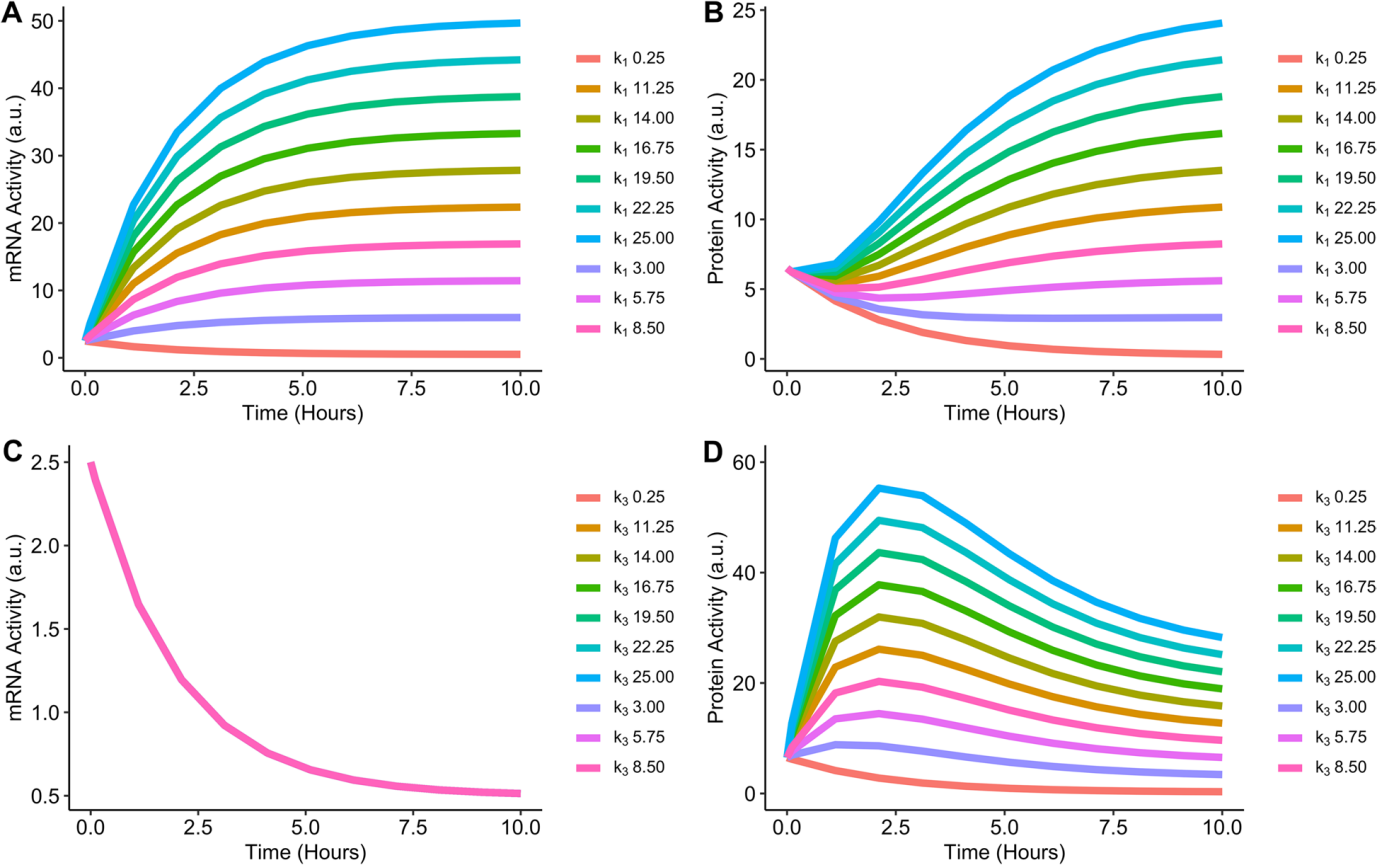
Change in mRNA (**A** and **C**) and Protein (**B** and **D**) activity due to change in ${k}_{1}$ and ${k}_{3}$ values. Initial values for the simulation are as follows: ${k}_{1}$ and ${k}_{3}$ = 0.25, ${k}_{2}$ and ${k}_{4}$ = 0.5, $mRNA(0)$ = 2.5 and $Protein(0)$ = 6.5

Figure 3 visualises the impact on changing the values of ${k}_{1}$ and ${k}_{3}$ on mRNA and protein activity.

Changes in ${k}_{1}$ have an impact on both mRNA and protein activity (Figure 3, A and B). However, changes in ${k}_{3}$ do not change the mRNA activity (Figure 3C).

For model 1, if the only observed output is mRNA, then the model is structurally unidentifiable. This outcome is visualised in Figure 3C, where if only mRNA is observed, modifying the parameter ${k}_{3}$ does not impact mRNA activity. Therefore for those wishing to utilise model 1, where mRNA alone is observed, any of the values in Figure 3C can be used for ${k}_{3}$ and the mRNA activity over time will be identical. This ultimately reduces the reliability and confidence in the estimated optimal value for the parameter ${k}_{3}$.

While this result could feasibly be determined based on the ODE system of model 1, the main goal was to visually showcase an unidentifiable parameter compared to an identifiable parameter. Model 1 with only protein observed is still structurally identifiable. Therefore, the decision was made to utilise model 1 to showcase model simulation for an unidentifiable parameter.

### Model 2

#### Structural identifiability results

Based on the system of ODEs and that all of the states are observed, applying the EAR approach, model 2 is structurally (locally) identifiable (at least).

#### Parameter estimates

The estimates were obtained using a nonlinear least square solver in *MATLAB*. Since ${D}_{r}$ and $GF$ are assumed to be known, the values of ${D}_{r}$ and $GF$ are estimated to be 0.017 hr^-1^ and 0.131 mM respectively. While the original study does provide values for ${D}_{r}$ and $GF$, during the analysis the use of the original values (0.033 hr^-1^ for ${D}_{r}$ and 7mM for $GF$) would often result in negative predictive values for glucose concentration. For demonstration purposes, the decision was made to re-estimate these parameters and subsequently assume they are known a *priori* . It is important to note that this analysis is based on the ODE system only unlike the original paper which is based on both the ODE system and the intracellular metabolic model.

**Fig. 4:**
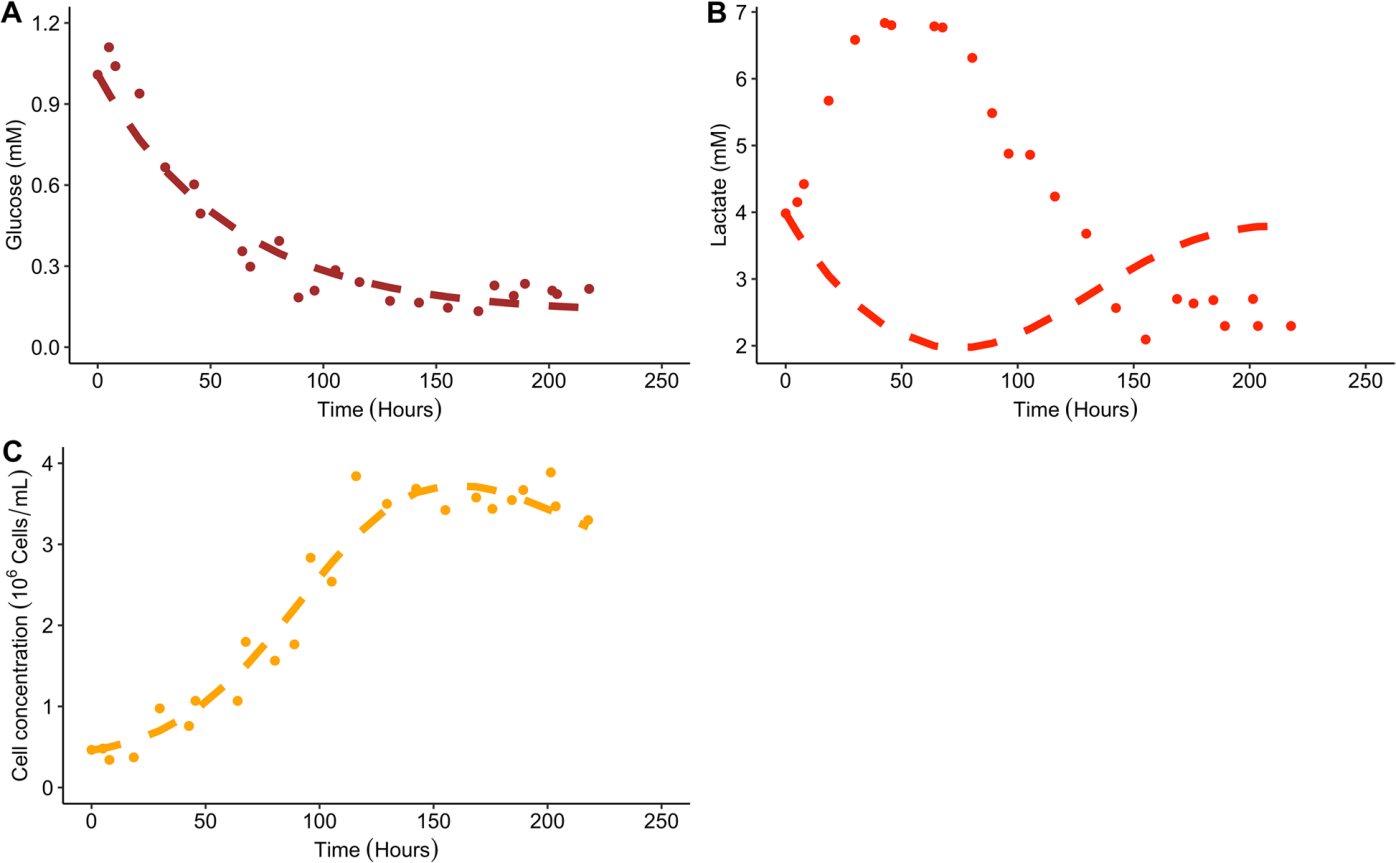
Predictions vs observations for glucose (**A**), lactate (**B**) and cell concentration (**C**) using the estimates summarised in Table 1. Dashed lines represent the model simulation whereas the dots represent the extracted experimental data

Figure 4 visualises the predictions vs observations obtained for model 2:

Figure 4A and Figure 4C show that the model predictions do follow the trajectory of the observed glucose and cell concentrations. Figure 4B shows that the model predictions are not able to capture the trajectory of the lactate data.

The estimates along with the 95% CI are summarised in Table 1

**Table 1:** Parameter estimates and 95% CIs for model 2 case 1

Parameters	Value	95% CI Lower bound	95% CI Upper bound
${r}_{glu}$((pmol/cell)/hr)	0.032	Infinite	0.228
${r}_{lac}$((nmol/cell)/hr)	0.020	0.010	0.029
${\mu }_{max}$(hr^-1^)	0.078	0.056	0.368
${km}_{glu}$(mM)	0.221	0.022	3.265
${ki}_{lac}$((mM)^2^)	10.954	1.890	36.099

Summary of parameter estimates and 95% CIs for model 2 case 1. Values are displayed to three decimal places

#### Practical identifiability analysis

##### Case 1: ${r}_{glu}$, ${r}_{lac}$, ${\mu }_{max}$, ${km}_{glu}$, ${ki}_{lac}$ are unknown

**Fig. 5:**
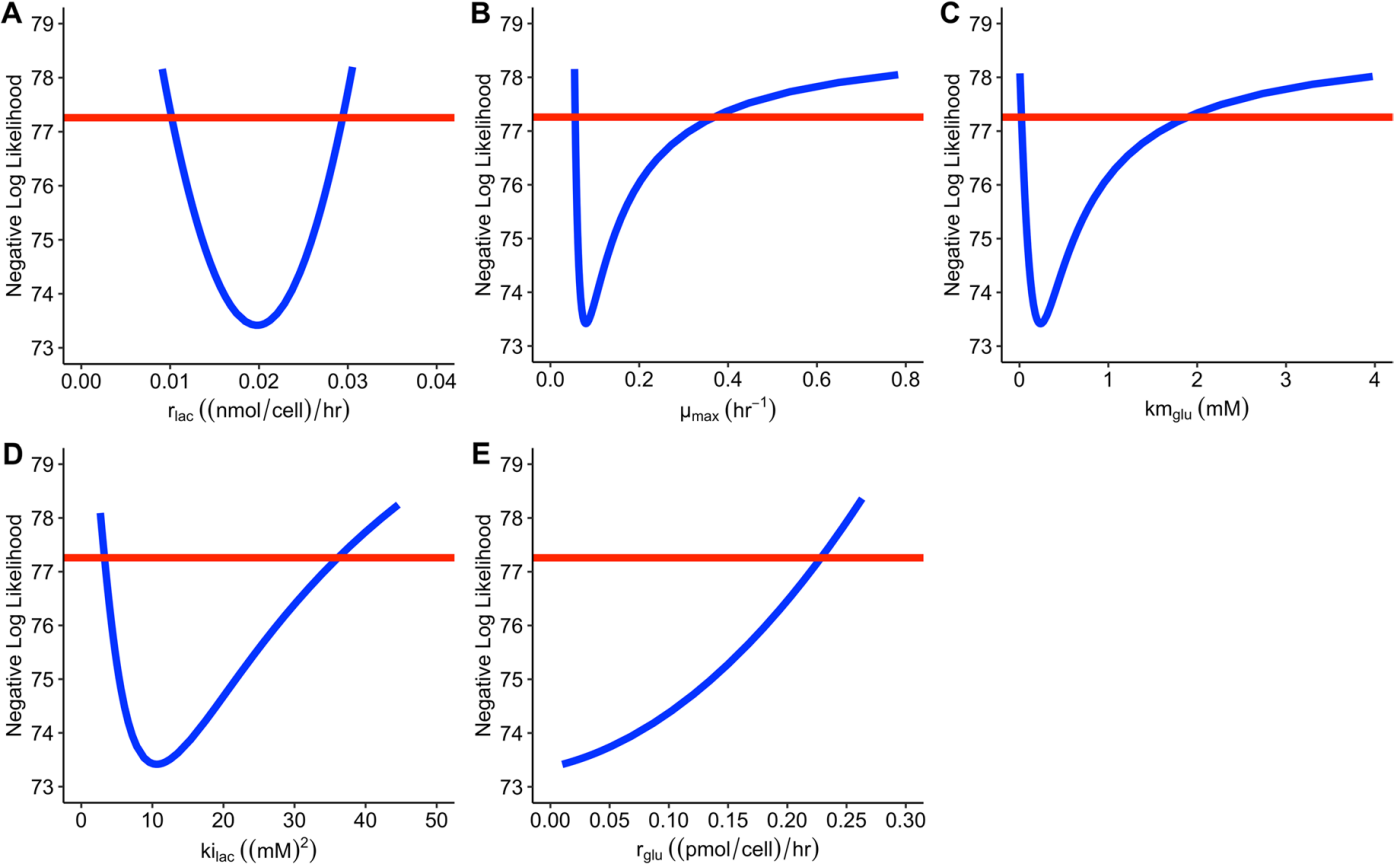
Profile likelihood for model 2 case 1. The Red line depicts the 95% CI threshold which is at 77.259. The Blue line depicts the estimated values for each of the parameters of interest. The 95% CI for each parameter can be found in Table 1

The profile likelihood plots for all the unknown parameters in model 2 is presented in Figure 5:

The parameter ${r}_{glu}$ is the only parameter in model 2 case 1 where the 95% CI threshold is not crossed for lower values. In Figure 5D, the parameter ${r}_{glu}$ does cross the threshold at 0.228 (pmol/cell)/hr (Table 1). Based on this result, model 2 case 1 is practically unidentifiable.

##### Case 2: ${r}_{glu}$ is known

This practical identifiability outcome is only achieved when ${r}_{glu}$ (assuming only one parameter can be measured) is known. ${r}_{glu}$ is assumed to be 0.032 (pmol/cell)/hr. Table 2 provides the new 95% CIs for model 2 case 2:

**Table 2:** 95% confidence intervals for model 2 case 2

Parameters	Lower bound	Upper bound
${r}_{lac}$((nmol/cell)/hr)	0.010	0.030
${\mu }_{max}$(hr^-1^)	0.056	0.333
${km}_{glu}$(hr^-1^)	0.020	1.708
${ki}_{lac}$((mM)^2^)	3.397	37.280

Summary of the 95% CI for model 2 case 2. Values are displayed to three decimal places

Note that the parameter estimates are the same as the estimates in Table 1.

**Fig. 6:**
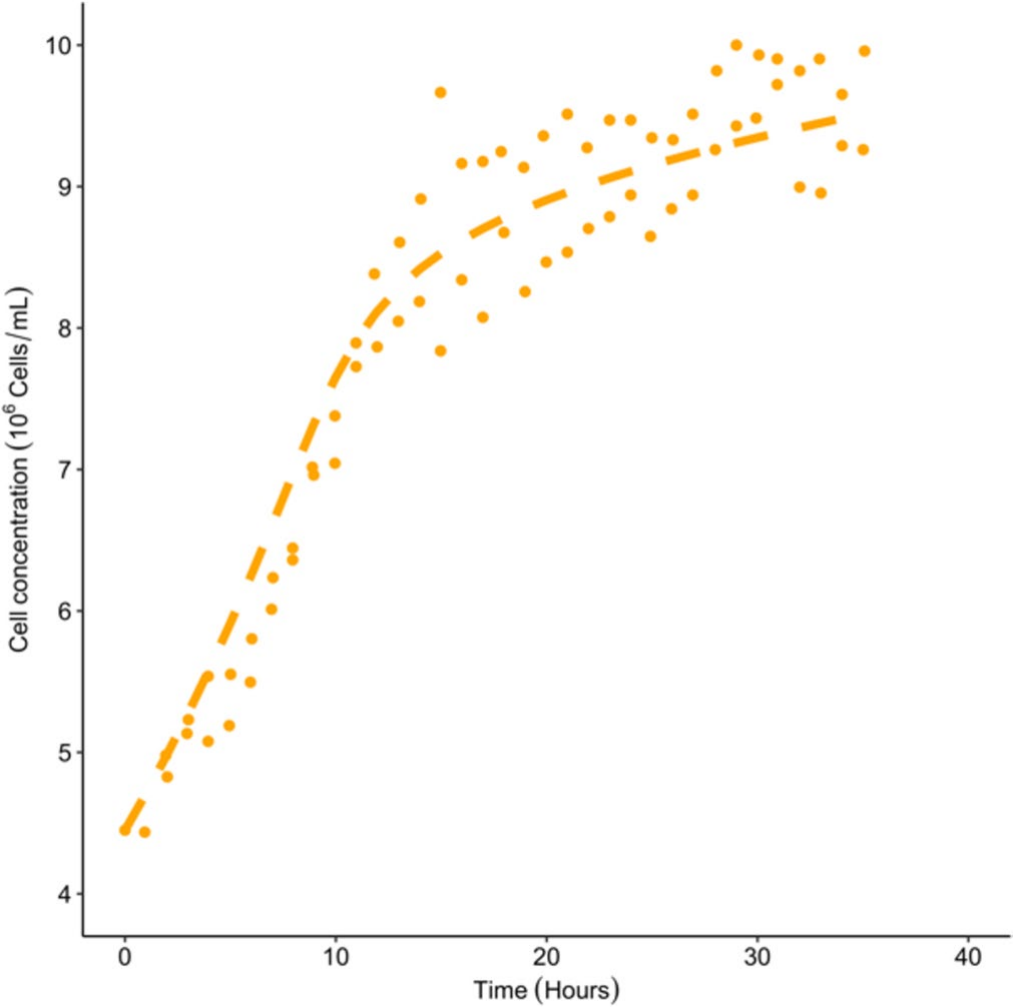
Predictions vs observations for cell concentration using the estimates summarised in Table 3. Dashed lines represent the model simulation whereas the dots represent the extracted experimental data

In Table 2, all of the parameters have values which cross the 95% CI threshold at the lower and upper bounds. Figure 6C, and Table 2 shows ${\mu }_{max}$ crossing the upper 95% CI threshold at 0.333 hr^-1^, which is lower than in model 2 case 1 (Table 1). The 95% CI for the parameter ${r}_{lac}$ is the same in Tables 1 and 2 (0.010 – 0.030). Based on these results model 2 case 2 is practically identifiable.

### Model 3

#### Structural identifiability results

Based on the system of ODEs and that $X$ is observed, applying the EAR approach, model 3 is structurally (locally) identifiable (at least).

#### Parameter estimates

Figure 6 visualises the predictions vs observations obtained for model 3:

The model is able to simulate the general logarithmic pattern experienced by the extracted cell concentration data, including the steady increase in cell concentration during the first 12 hours (Figure 6).

The estimates along with the 95% CI are summarised in Table 3.

**Table 3:** Parameter estimates and 95% CIs for model 3 case 1

Parameters	Value	Lower bound	Upper bound
${r}_{g}$(hr^-1^)	0.057	0.048	0.050
$a$((cellsx10^6^/mL)^-1^)	2.6	6.804	Infinite
$b$(cellsx10^6^/mL)	3.4	3.609	3.894
${r}_{d}$(hr^-1^)	0.005	0.011	0.015

Summary of parameter estimates and 95% CIs for model 3 case 1. Values are displayed to three decimal places

#### Practical identifiability analysis

##### case 1:${r}_{g}$, ${r}_{d}$, $a$, $b$ are unknown

**Fig. 7:**
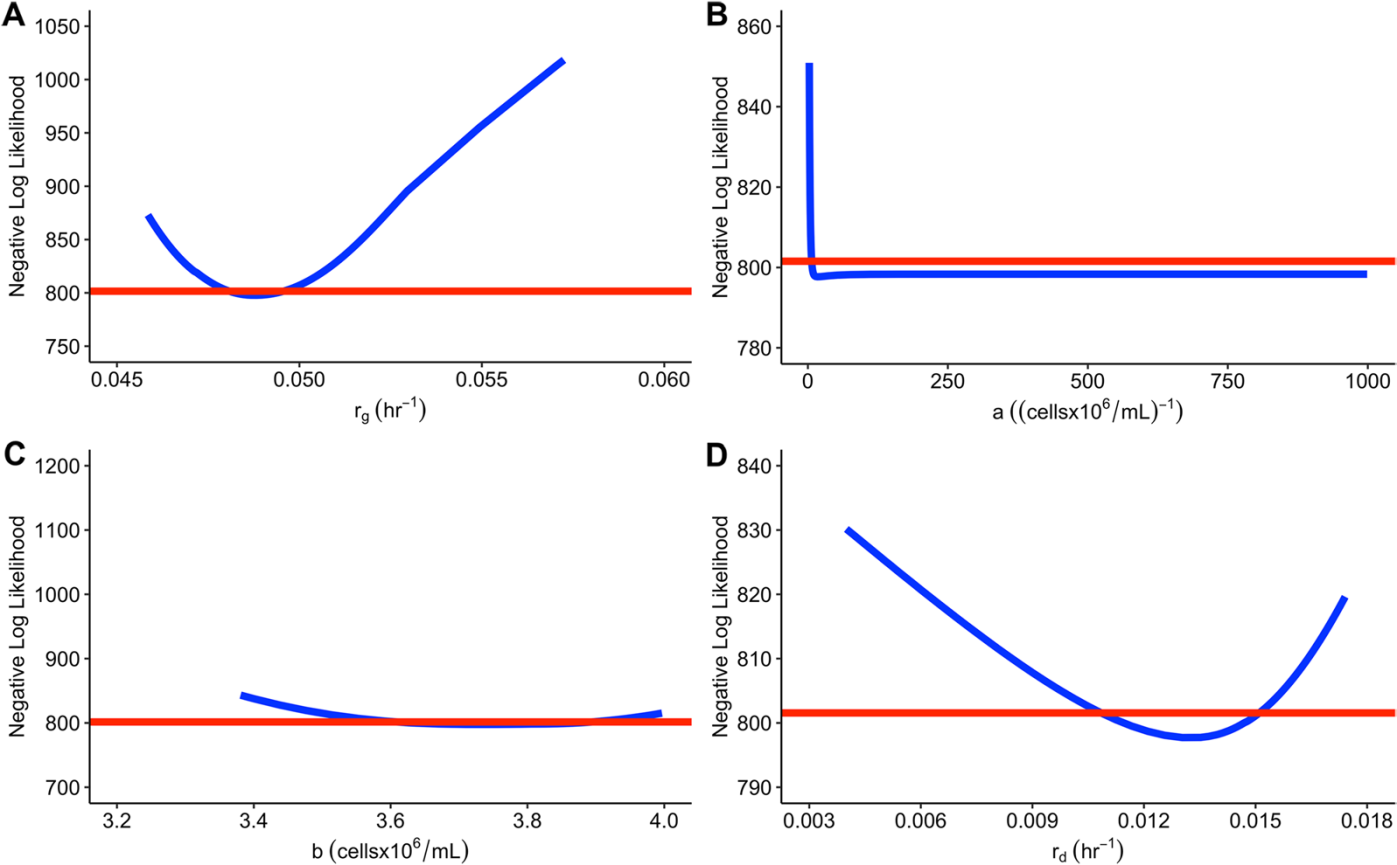
Profile likelihood for model 3 case 1. The red line depicts the 95% CI threshold which is at 801.553 and the blue line depicts the estimated values for each of the parameters of interest. The confidence intervals for each parameter can be found in Table 3

The profile likelihood plots for all the unknown parameters in model 3 is presented in Figure 7:

The parameter $a$, is the only parameter in model 3 case 1 which does not cross the 95% CI threshold at both sides (Figure 7). In Table 3, the parameter $a$ does cross the threshold at 6.804 ((cellsx10^6^/mL)^-1^). Based on these results, model 3 case 1 is practically unidentifiable.

##### case 2: $b$ is known

This practical identifiability outcome is only achieved if $b$ (assuming only one parameter can be measured) is known. $b$ is assumed to be 3.4 cellsx10^6^/mL. Table 4 provides the new 95% CIs for model 3 case 2:

**Table 4:** 95% confidence intervals for model 3 case 2

Parameters	Lower bound	Upper bound
${r}_{g}$(hr^-1^)	0.049	0.050
$a$((cellsx10^6^/mL)^-1^)	5.283	10.806
${r}_{d}$(hr^-1^)	0.015	0.018

Summary of the 95% CI for model 3 case 2. Values are displayed to three decimal places

Note that the parameter estimates are the same as the estimates in Table 4. All of the parameters cross the 95% CI threshold for both lower and higher values . The parameter $a$ now has a shorter 95% confidence interval (Table 4) compared to model 3 case 1 (Table 3). Based on these results model 3 case 2 is practically identifiable.

## Discussion

Models that are structurally and practically identifiable enable researchers to have increased confidence in the parameter estimates that are obtained during the parameter fitting process. An unidentifiable model can still obtain a close fit to the experimental data, however the estimates obtained from the structurally or practically unidentifiable parameter can take on various values without changing the predicted output and thus obtaining the same outcome.

This guide utilises two different methods to perform the structural identifiability analysis. The Taylor series approach is a method that can be applied in any software/tool that can handle symbolic computations (i.e., that can manipulate mathematical expressions). However, for more complex models, the coefficients can become challenging to solve [14]. Moreover, the Taylor series approach can become time exhaustive for complex models. The EAR approach allows users to simply define the system of ODEs, declare the observed states and identify which of the parameters are known. The tool will indicate whether or not a model is identifiable and in the event that the model is unidentifiable, the user can further explore how many parameters are needed in order to make the model identifiable and which parameters are unidentifiable. The EAR approach offers a convenient way for modellers to understand the structural identifiability of their models. However, the EAR approach requires a license for *MATHEMATICA* and for complex models may require simplification of the original model, as in the case for model 3. In addition to this, in instances where the model may be too complex, the EAR approach may indicate that a model is unidentifiable when in reality, the model is identifiable. Therefore it is important to compare the results obtained using multiple approaches in order to fully evaluate the structural identifiability of a model.

For models that are structurally unidentifiable, a model redesign, measuring more outputs, or knowledge of some (or in some cases all) of the unidentifiable parameters can be incorporated in order to make a structurally unidentifiable model identifiable (Figure 1).

The results for models 2 and 3 highlight the fact that just because a model is structurally identifiable this does not mean that a model is also practically identifiable. Moreover, the analysis of both models 2 and 3 emphasise two important points. For model 2, even if a model is both structurally and practically identifiable (if ${r}_{glu}$ is known and all parameter estimates remain the same, which will generate the same predictions vs observations as Figure 4), this does not mean that the model is necessarily able to capture all of the data as evidenced in Figure 4B. In this instance the model was not able to fully capture the changes in lactate concentration. This would suggest that the model although structurally and practically identifiable, would need to be redesigned in some appropriate way in order to simulate the observed changes in lactate concentration. The analysis of model 3 indicates that, even if a structurally identifiable model does produce a good fit to the data, this does not necessarily mean that the model is practically identifiable as parameter $a$ is practically unidentifiable for model 3 case 1. All three processes (structural and practical identifiability analyses and model fitting) are required in order to produce a model that is considered to provide a robust representation of the data.

As mentioned in the introduction section, possible methods to make structurally unidentifiable models identifiable include, where practically feasible, the possible inclusion of additional observed outputs as well as the potential re-design or reparameterisation of the ODE system. Structural identifiability analysis should be performed prior to experimental design. In Figure 1, one of the methods to combat practical unidentifiability is to increase the data streams available through wider experimentation or the collection of outputs of additional state variables. An increase in sample size will result in a narrower 95% CI which can make infinite intervals become finite. Another option which was used for both models 2 and 3 was to assume that one of the unknown parameters was known. This change alters the degrees of freedom which also influences the ${\chi }^{2}$ value. However, unlike structurally identifiability where it is the unidentifiable parameters that need to be known, for practical identifiability, knowledge of practically identifiable parameters can also influence the practical identifiability of a model. This could possibly be linked to the dynamic structure of the ODE system and how certain parameters are related to each other, which can be viewed through the analysis of the Taylor series coefficients. It is therefore important to analyse the practical identifiability results based on different scenarios pertaining to knowledge of different parameters to assess which case is the optimal set up.

The main limitations with practical identifiability analysis are that it is very sensitive to the data used and the primary parameter estimations. The data that were used to perform the PIA for both models 2 and 3 were extracted from the relevant studies in the literature, moreover one model was made to simulate multiple observations. Therefore, it is possible that with actual data the results may be different. It is also plausible that some of the estimates obtained are not feasible for the system that the model is attempting to represent. However, this further drives the need that awareness of structural and practical identifiability is crucial to allow more users to freely access and evaluate their models. Finally, a structurally and practically identifiable model does not necessarily indicate that the identified model is the best model for the data prediction. Further analysis would be necessary to identify which model a user should ultimately choose to best represent the data.

## Conclusions

In conclusion, models from bioengineering were analysed for their structural and practical identifiability in an accessible manner which will hopefully incentivise more users to also utilise these tools and assess their models to support experiment design and robust parameter estimation. Further research will include analysing the practical identifiability of these models to identify whether optimal experimental conditions can be set up for model development, robust estimation of unknown model parameters and output optimisation.

## Data Availability

All relevant codes and data that were used for the study are available in the SIA and PIA of bioengineering models Github repository, https://github.com/LindaWanika/SIA_and_PIA_of_bioengineering_models
